# Intake of Sugar Substitute Gummy Candies Benefits the Glycemic Response in Healthy Adults: A Prospective Crossover Clinical Trial

**DOI:** 10.3390/gels8100642

**Published:** 2022-10-10

**Authors:** Dan Gan, Minjun Xu, Ling Chen, Shaohua Cui, Changyong Deng, Qian Qiao, Ruimiao Guan, Fang Zhong

**Affiliations:** 1 Sirio Pharma Co., Ltd., Shantou 515000, China; 2 State Key Laboratory of Food Science and Technology, Jiangnan University, Wuxi 214122, China; 3 Science Center for Future Foods, Jiangnan University, Wuxi 214122, China; 4 School of Food Science and Technology, Jiangnan University, Wuxi 214122, China; 5 International Joint Laboratory on Food Safety, Jiangnan University, Wuxi 214122, China

**Keywords:** gummy, maltitol, erythritol, glycemic index, glycemic load

## Abstract

Sugar reduction in food has attracted great health concerns worldwide. Gummies have been one of the most popular and highly favored candies due to their chewable properties, simplicity to swallow, and delicious taste. The general perception is that gummies raise blood sugar levels, but the truth is that gummies with the right formula can control glycemic response. The purpose of this study is to investigate the effects of the gummy dosage form and sugar types on the glycemic response control. Maltitol and erythritol as sweetener alternatives were applied in gummy candies (total and partial sugar substitutes gummy, T-SG and P-SG), with sucrose-based gummies used as comparisons (CG). A prospective crossover study was then conducted on 17 healthy adults. The effects of different types of gummies on glycemic response in healthy adults were evaluated on the basis of the participants’ glycemic index (GI) and glycemic load (GL) values. Every three-day interval, participants took CG, P-SG, T-SG, and glucose solution, respectively, and the theoretical glucose conversion content was kept the same in all groups for each trial. Each participant performed four tests with each sample and recorded the changes in blood glucose after food consumption. It was found that all three types of gummies slowed down subjects’ glycemic response when not taken in excess, and the improvement effect was in the trend of T-SG > P-SG > CG. Both P-SG and T-SG were low-GI candies (54.1 and 49.9). CG that was not consumed in excess of 17.2 g had a high GI (81.9) but a low GL (<10). Texture analysis and in vitro digestion were used to explore the effect of gummy matrix on glucose release. T-SG and P-SG retained a higher hardness and were less hydrolyzed to release glucose during digestion compared with CG. Additionally, experiments have revealed that gummies can reverse the poor glucose tolerance in women. In conclusion, gummies are a good carrier for dietary supplements due to their sustained-release characteristic of available carbohydrates and provide healthier options for people in control of glucose homeostasis.

## 1. Introduction

Carbohydrates are one of the essential nutrients and sources of energy for the human body. According to the “Dietary Guidelines for Chinese Residents (2019)” and the “Dietary Guidelines for Americans (2015–2020)” released by the Office of Disease Prevention and Health Promotion (ODPHP), the daily carbohydrate consumption should reach 60% to 70% of the total daily caloric intake [1,2]. However, excessive glucose intake or impaired glucose metabolism usually presents a high blood glucose level. Long-term high blood glucose levels may induce obesity, cardiovascular disease, diabetes, and other diseases, which are all serious threats to human health [3]. Moreover, studies have also demonstrated that obesity, atherosclerosis, and diabetes will further lead to glucose metabolism disorders, raising the blood glucose level, which forms a vicious circle [4,5]. Therefore, maintaining blood glucose homeostasis is vital to health.

The glycemic index (GI) and the glycemic load (GL) are two parameters used for evaluating the quality and quantity of carbohydrates-containing food. The GI measures the ability of the available carbohydrates in foods to increase blood glucose, usually expressed as a percentage of the glucose [6]. Thus, the GI of glucose is defined as 100, and foods with a GI lower than 55 are considered as low-GI foods, whereas those with a GI between 55 and 70 are medium-GI foods, and those with a GI exceeding 70 are high-GI foods [4]. It has been found that type 2 diabetes patients consuming a low-GI diet have better performance in controlling the glycated hemoglobin level and fasting blood glucose level compared to those with high-GI diet [7]. Therefore, instead of a high-GI diet, a low-GI diet has been recommended as a dietary intervention to manage hyperglycemia or obesity [8]. However, the GI only indicates the glycemic response to the carbohydrates in various foods without considering the amount of food consumed. The GL of the food is the product of its GI and its available carbohydrate content, evaluating both the quantity and the quality of carbohydrates. Foods with a GL lower than 10 are considered as low-GL foods, those between 11 and 19 are medium-GL foods, and those higher than 20 are high-GL foods [4]. Overall, GL considers every dietary component as a whole, providing a more real-life picture of how a dish will affect your blood glucose levels.

It is worth noticing that both GI and GL are calculated based on the amount of available carbohydrates. Available carbohydrate is defined as the content of carbohydrate that can produce glucose and be absorbed by the small intestine. In general, a higher carbohydrate content causes a greater response to blood glucose levels. However, aside from available carbohydrates, there are numerous other food factors that can affect GI and GL values, one of which is gelling properties of the food. The chewiness and swallowability of gummies have made them popular among consumers due to the versatility of hydrocolloids in forming specific dosage forms [9]. Hydrocolloids used in gummies can slow down mixing and mass transfer processes and alter the release of flavors or nutrients by increasing the viscosity of the gastrointestinal fluids in the stomach or small intestine, because of their thickening and gelling abilities [10]. Ordinary gummies contain large amounts of sweeteners such as sucrose, glucose syrup, and gelatin, as well as seasoning, which is crucial for consumer acceptance due to their sweetening ability and influence on the product’s texture [11,12]. Sucrose is the most commonly used sweetener, but it has gradually been replaced by artificial sweeteners because of its high glycemic index. Although artificial sweeteners almost have no calories, there is still a concern for potential safety hazards [13]. Maltitol and erythritol are considered as ideal alternatives not only because of their pure sweetness, but also due to the limited hydrolyzation by the human enzyme system of them, which induces little effect on glucose metabolism [14,15]. Recent research has reported that the composition and concentration of sugar alcohols have an impact on the texture and gelling properties of hydrogels [16], and the change in gel texture would further affect digestion and glucose release in the digestive tract [17]. As far as we know, it is still unclear how maltitol and erythritol affect the structure of gummies and how they affect humans’ blood sugar homeostasis.

This study aimed to explore the effect of sugar alternatives and dosage form of gummies on glycemic response. It was discussed above that GI and GL values are highly dependent on available carbohydrates, so, in this study, we maintain consistent carbohydrate levels across samples (the same theoretical glucose conversion content). Sugars in total and partial sugar substitutes gummies, T-SG and P-SG, were substituted by erythritol and maltitol, while sucrose-based gummies were used as comparisons (CG). All the gummies were tested on healthy adults. The glycemic index (GI) and glycemic load (GL) values were then determined to assess the glycemic response to various gummy samples. Texture analysis and glucose release rate of gummies during in vitro digestion were carried out to probe the underlying mechanisms [18]. The results would provide additional health information of low-GI gummies related to weight management and chronic disease prevention. This study also provides a reference for the health of normal people ingesting glycemic control products.

## 2. Results

### 2.1. Glucose Tolerance Test of Gummy Candies

**Figure 1:**
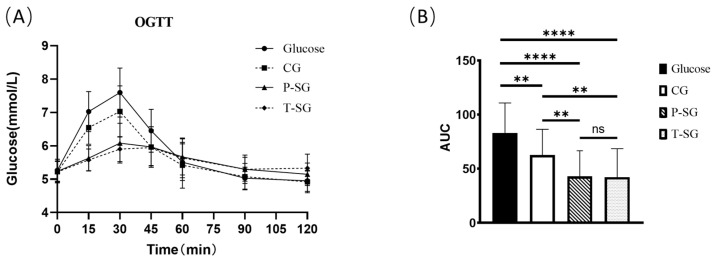
OGTT curve (**A**) and OGTT AUC (**B**) after consumption of 10 g of glucose or three types of nutritional gummies providing equivalent amounts of available carbohydrates. Intragroup comparisons were made using the paired Student’s *t* test. ****, *p* < 0.0001; **, *p* < 0.005; ns, no significant difference. T-SG: total sugar substitutes gummy; P-SG: partial sugar substitutes gummy; CG: comparison gummy (sucrose-based gummy).

The curve and area under curve (AUC) of oral glucose tolerance test (OGTT) demonstrated a significantly improved glucose tolerance in all the gummy groups compared to the glucose group. Statistically significant differences were also observed between groups with CG and P-SG/T-SG (*p* < 0.05). Moreover, there was no significant difference between the P-SG and T-SG groups. The OGTT curve was steadier in the P-SG and T-SG groups than in the CG and glucose groups (Figure 1).

Blood glucose appeared to rise 15 min after food consumption. The 15 min blood glucose levels in the CG, P-SG and T-SG were significantly lower than that in the glucose group. Furthermore, lower blood glucose levels were found in the P-SG and T-SG groups compared to the CG at 15 min. The blood glucose peak of the glucose, CG, and P-SG groups appeared at the 30 min mark after food consumption. However, the blood sugar peak of the sugar-free group appeared at the 45 min mark after food consumption. The peak blood glucose levels of all three gummies were significantly lower than glucose. Meanwhile, the peak blood glucose levels in the P-SG and T-SG groups were also lower than that in the CG group. The blood glucose levels at the 15 min mark and the 30 min mark were not different between the P-SG and T-SG group. At the 60 min mark, the blood glucose levels of the glucose group and the CG group were lower than the P-SG group and the T-SG groups. At the 90 min mark, the blood glucose levels of the P-SG and the T-SG groups returned to baseline levels (levels at the 0 min mark). However, the blood glucose levels of the CG and the glucose groups at the 90 min mark were significantly lower than their baseline blood glucose levels (at the 0 min mark). At the 120 min mark, there was no significant difference in blood glucose levels of the P-SG group and the T-SG group from the baseline level (at the 0 min mark). However, the blood glucose levels of the CG and the glucose groups at the 120 min mark were significantly lower than their baseline blood glucose levels (at the 0 min mark) (Shown in Table S2).

GEE analysis results showed that compared with glucose, blood glucose concentration was significantly lowered in the P-SG group and T-SG group at the 15 min, 30 min, 45 min, 60 min, and 120 min marks, except for the 90 min mark (*p* < 0.001). In addition, the peak of blood glucose concentration appeared at the 30 min mark. Although there was no significant difference, the blood glucose level at the 90 min mark was slightly higher than at 120 min (Table 1).

### 2.2. Glycemic Index (GI) and Glycemic Load (GL) 

The Glycemic Index (GI), Glycemic Load (GL), and the classification of the three types of gummies are illustrated in Table 2. Both P-SG and T-SG were low-GI types of food. Although the CG is classified as a high-GI food, it was also found to be a low-GL food when taking the carbohydrate quantity into consideration. As a matter of fact, when the intake amount of the CG was kept within six gummies, the CG’s GL classification remained low (GL < 10), while the P-SG and T-SG products allowed for a much higher intake allowance.

### 2.3. Differences in Glucose Tolerance between Male and Female

**Figure 2:**
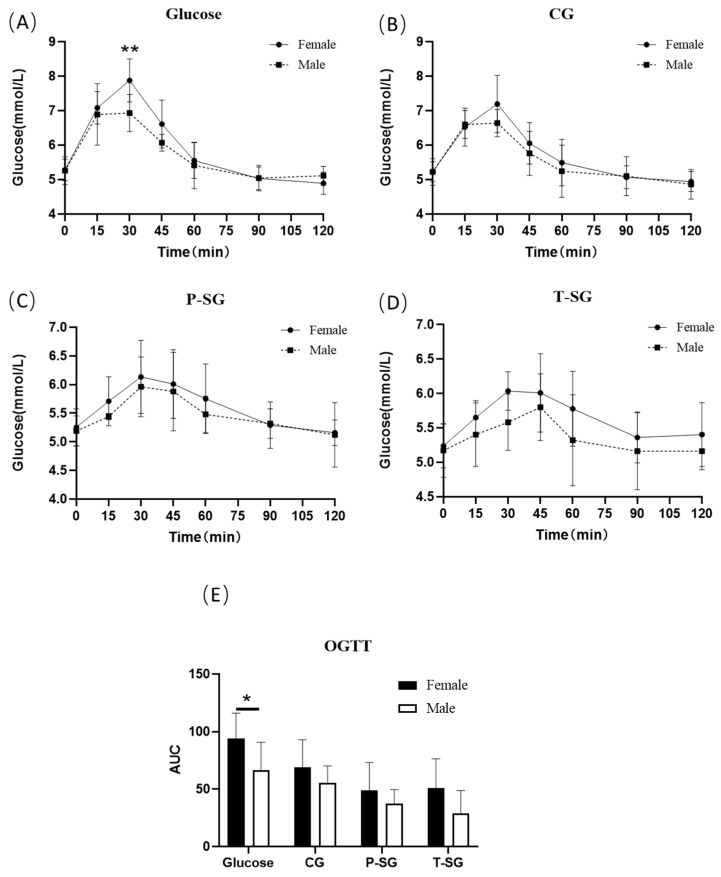
OGTT curve (**A**–**D**) and OGTT AUC (**E**) of males and females after consumption of a 10 g glucose bolus or three types of nutritional gummies providing equivalent amounts of available carbohydrates. * *p* < 0.05; ** *p* < 0.01. OGTT: oral glucose tolerance test.

The results of OGTT indicated a significantly improved glucose tolerance in males compared to females with respect to glucose intake. However, in the gummy groups, there was no statistically significant difference between genders. Research data suggested that gummy format counteracted sugar-induced glucose intolerance in females (Figure 2).

### 2.4. Mechanical Properties of Gummy Candies

Table 3 shows the TPA results of gummies with CG, P-SG, or T-SG. Despite the relatively higher springiness, cohesiveness, and resilience of CG, it had a significantly lower hardness than P-SG and T-SG. Additionally, CG had a significantly lower gumminess and chewiness compared with other two gummies.

### 2.5. Glucose Release of Gummy Candies during In Vitro Digestion

**Figure 3:**
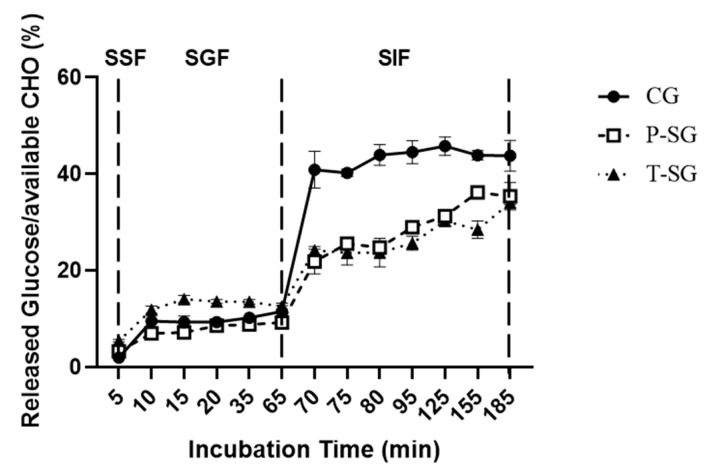
Glucose release from CG, P-SG, and T-SG during SSF (first 5 min), SGF (5–65 min), and SIF (65–185 min) incubation. CHO: Carbohydrate. SSF: simulated salivary fluid; SGF: simulated gastric fluid; SIF: simulated intestinal fluid.

Based on Figure 3, only a small amount of glucose was released after 5 min SSF incubation for all gummy samples. A relatively higher rate of glucose release was found in T-SG in the gastric fluid, probably due to its less cohesiveness property. It was reported that gels with low cohesiveness were considered brittle gels, and they tended to break into smaller pieces when milled in the stomach [17]. In the intestinal phase, a significantly increased glucose release rate of CG was observed, but no significant difference was found between P-SG and T-SG at the end of SIF incubation. The hardness of CG was significantly lower than that of P-SG and T-SG, which meant that CG was more easily broken into pieces during long shearing sessions. As a result, glucose was more easily released outside.

## 3. Discussion

The results showed that the P-SG and T-SG groups elicited better glucose tolerance. Both P-SG and T-SG were low-GI foods. All three types of gummies can slow the post-prandial rise in blood glucose and were low-GL foods when not taken in excessive amounts. Since the available carbohydrates were same in each group, the difference in GI values is likely due to texture differences and subsequent digestive behavior differences among different kind of gummies. Tharakan et.al. reported that simulating intestinal digestion, in which thickening agents (guar gum) were added to the simulated fluids, reduced the rates of glucose release [18]. It has been explained by McClements that gummies contain hydrocolloids that may increase the viscosity of the gastrointestinal fluids, thereby slowing the mixing and mass transfer process, and altering the flavors or nutrients release [19]. Accordingly, gummy dosage forms in this study significantly improved blood glucose control. Because each kind of gummy was made with the same gum base, their different texture properties may be due largely to the differences in sweetener used in the gummy. Although some research reported that sugar/sweeteners type did not significantly influence textural properties [20], other research discovered that the type of sugar used would affect hardness levels [21]. Gummy candy containing more sucrose than other sweeteners had weak gel characteristics possibly because of the hindrance effect of sucrose recrystallization at the aging period [21]. This is confirmed by our study, where we found CG showed a lower hardness, gumminess, and chewiness as compared to P-SG and T-SG, which indicated that it would be easier to break into pieces and release sugar during digestion. The results of the in vitro release study that CG did present a significantly higher release of glucose than P-SG and T-SG supported above assumptions.

In addition to the gelling properties of different types of gummies, the distinction in GI value of the sweeteners within them could also affect blood glucose levels. Erythritol is a four-carbon molecule. Approximately 10% of the ingested erythritol can escape to the large intestine, and, meanwhile, it has a negligible effect on the glycemic response. Because of this unique metabolic characteristic, it is frequently used in common and functional foods for people with special health demands, such as diabetic and obesity patients. Livesey’s research revealed that the glycemic index and insulin index of erythritol are 0 and 2, which is much lower than that of xylitol [22]. According to the reports of the European Medical Research Center [23]: Erythritol can react with hydroxyl radicals as a free radical scavenger and metabolic inhibitor to form erythrose and erythrulose, preserving the function of endothelial cells. Maltitol is a disaccharide polyol produced by starch hydrolysis and must undergo hydrolysis to become glucose and sorbitol before being absorbed. However, it was found that α-1,4-glycosidase in the human digestive tract could not efficiently decompose the maltitol into glucose. The above findings suggest that the exposed available carbohydrates in P-SG or T-SG would be slightly hydrolyzed by digestive enzymes, releasing a lower amount of glucose. As discussed above, the results of the in vitro digestion experiment were consistent with those obtained in the clinical study. Gummies with low GI values (P-SG and T-SG) demonstrated a significantly lower AUC than gummies with a high GI value (CG), even though the amount of available carbohydrate was the same in all cases. In the present study, we proved that the texture properties of P-SG and T-SG, as well as their limited digestive hydrolysis, contribute to the low GI value. Gummy candies containing erythritol and maltitol instead of sucrose have a low GI value, providing a theoretical basis for the implementation of erythritol and maltitol in the manufacturing of food supplements for diabetics.

Furthermore, the present study also revealed that women tend to have a significantly lower glucose tolerance than men after ingesting glucose dissolved in water. In previous reports, it has been demonstrated that the correlation between visceral fat content and glucose homeostasis in women is significantly higher than that in men. Compared with men, healthy women have less skeletal muscle mass, more adipose tissue mass, more circulating free fatty acids (FFA), and more intracellular lipid contents, which all promote insulin resistance in women [24]. Sicree et al. reported that, in the Australian population, women have lower fasting blood glucose levels and higher AUC after OGTT than men [25]. Additionally, sex hormones also play a vital role in maintaining glucose homeostasis. The beneficial effects of endogenous testosterone on male glucose homeostasis have also been proven [26,27]. The aforementioned findings are consistent with this study’s results. Nevertheless, the results delineated that there was no statistically significant difference in terms of glucose tolerance between men and women after ingesting P-SG and T-SG. It can be explained by the insulin-independent characteristic of these low-GI gummies, suggesting that low-GI foods are more conducive to maintaining female glucose homeostasis.

Interestingly, this study results also demonstrated that the difference in glucose tolerance between women and men in the glucose group was reduced after consuming CG (*p* > 0.05), and there was no significant difference between genders at 30 min after ingestion. This may be related to chewing, which is the first step of mechanical digestion and facilitates the mixing of salivary amylase with food. Previous studies have shown that more chewing increases the flow rate of saliva, which is positively correlated with the activity of salivary amylase-alpha. Chewing also enhances the secretion of GLP-1, a protein known to enhance glucose-stimulated insulin secretion [28]. Passeri’s research demonstrated that, in the obese population, women had a worse chewing function, which might be related to hyperglycemia and obesity [29]. The study performed by Sato et al. also proved that an increase of chewing time led to a reduction in blood glucose levels after breakfast and boosted insulin secretion [30]. Consistent with hypothesis, the in vitro digestion result showed that, in the oral phase, glucose release rate was slower in gummy form than in powder form. Additionally, digested total available carbohydrate was lower in gummy form. As a result, the gummy dosage form with its chewable characteristics is more likely to become a candy dosage form suitable for people with glucose metabolism disorders.

Some limitations in the current study were identified. Although this study achieved some meaningful and important results, only 17 participants were selected for the trials. Therefore, in the future, more participants need to be recruited for the trials to verify the conclusions of this study. On the other hand, all subjects in this study were given the same amount of gummy and glucose, rather than according to body weight. Lean body mass in males is usually heavier than in females, and the skeletal muscle is the most important organ of energy metabolism. It may also be the cause of the gender difference. Hence, in order to confirm the main cause of the difference in metabolism capacity between males and females, carbohydrates need to be assigned according to lean body mass in future studies. In this research, the available carbohydrates of all samples in all experiments were kept the same, indicating the absorbable glucose should be identical if no other influential factors were involved. Thus, the differences in blood glucose levels may be largely attributed to the different digestive release behavior of sweeteners from gummies. Although a TPA and an in vitro digestion study were conducted to evaluate the texture properties of gummies and the digestive release behavior, the specific mechanism behind how sugar type affects hydrogel structure and how the changes in gel structure influence enzymatic activity and glucose release during digestion remain unclear, which need to be investigated further.

Our study indicated that the P-SG and T-SG groups elicited better glucose tolerance. All the gummies were low-GL foods, and gummy format counteracted glucose intolerance in females. Furthermore, the P-SG and T-SG provided healthier options for people in control of glucose homeostasis. In general, sugar substitute gummy candies could be good potential food supplements for improving glycemia.

## 4. Conclusions

The three types of gummy candies used in the present study are all low-GL foods, which contribute to a reduced postprandial glycemic response when taken within the recommended dosage. Only the P-SG and T-SG were low-GI foods, and the CG was a high-GI food. Experimental results showed that, compared with high-GI food, low-GI gummies exhibited significantly improved postprandial glycemia, regardless of available carbohydrate levels. In part, this is due to the texture differences created by the sweeteners used in the gummies, which affects digestive behaviors and the subsequent glucose release after oral administration. Gummy candies with low GI values were proven to be healthier options for customers following weight management programs and for preventing chronic disease.

## 5. Materials and Methods

### 5.1. Clinical Study Design and Subjects

This prospective crossover study enrolled healthy adults and was conducted from July 2020 to September 2020. The inclusion criteria in brief were non-diabetic healthy young adults aged 22–39 years old, body mass index (BMI) within the range of 19–23.9 kg/m^2^, fasting glucose level ≤ 6 mmol/L, who were able to tolerate at least 10 h of fasting. The exclusion criteria were pregnancy and breastfeeding or subjects with known neoplastic, metabolic, digestive, endocrine, mental diseases, or taking medications affecting glucose tolerance; night-time or heavy physical workers or persons with insomnia, eating disorders, or those on a ketogenic diet within the last 6 months.

Informed consent was obtained from all the participants before the start of the study. The authors applied for human ethics approbation to the Chinese Ethics Committee of Registering Clinical Trials for this study. The number of the human ethics approval is ChiECRCT20200131.

### 5.2. Panel Demographics and Subjects Baseline Characteristics

**Figure 4:**
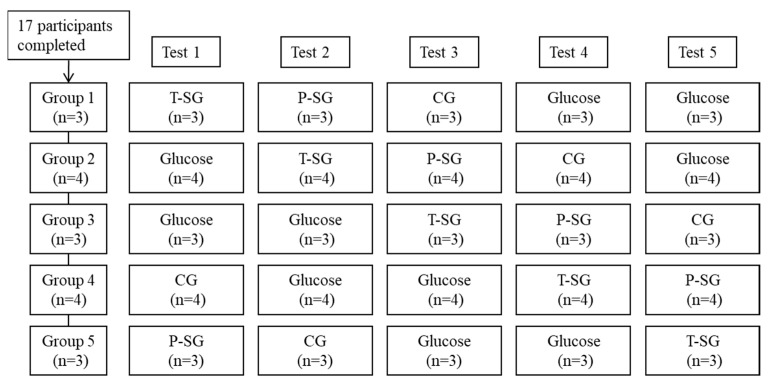
Flow diagram of subjects throughout the study. Tests days were five non-consecutive days with ≥72 h intervals. Final data collected for each food: CG (*n* = 17), P-SG (*n* = 17), T-SG (*n* = 17), Glucose (*n* = 34).

A total of 17 participants were enrolled. All the participants completed the experiment as required. The final per-protocol analysis sample therefore comprised 17 participants. The procedure that 17 participants (5 males and 12 females) underwent, two glucose tests and three sample gummies tests (CG, P-SG and T-SG), is displayed in Figure 4. To arrange the five tests in five groups in an orderly manner without missing any tests, we used the staggered method. The average age and BMI of males was 28.6 ± 4.3 years old and 21.3 ± 1.3 kg/m^2^, while the average age and BMI of females was 27.4 ± 3.1 years old and 22.2 ± 1.4 kg/m^2^, respectively. The fasting blood glucose was 5.3 ± 0.3 mmol/L in males and 5.2 ± 0.3 mmol/L in females. There were no significant differences in age, BMI, and fasting blood glucose between both genders at baseline (Table 4).

### 5.3. Procedures and Data Collection

The nutritional composition of gummy samples was assessed and presented in Table 5. Gummies tested in this research were plant-based gummies, so the carbohydrates% was around 70–100%. The sugars used in sucrose-based gummies (CG) were from glucose syrup, sucrose, and concentrated apple juice. In total and partial sugar substitutes gummies (T-SG and P-SG), sugars were replaced by maltitol syrup, erythritol, as well as some minimal sugar provided by concentrated apple juice; meanwhile, concentrated apple juice was further removed for T-SG (Table 5).

Tests were carried out on five non-consecutive days with intervals of at least 72 h between each testing day. The day prior to the experiment, a unified dinner was provided to the volunteers in a specified time range to control their protein and fiber intake. The participants were advised to avoid strenuous exercise, smoking, or alcohol consumption, and were instructed to fast for at least 10 h. Fasting glucose tests were performed twice with a 5 min interval. CG, P-SG, and T-SG with 250 mL of water and glucose solution (10 g of glucose dissolved in 250 mL of water) were consumed within 5 min. Each group received a different amount of gummy (Table 6, Portion weight, g) to ensure the amount of carbohydrates available (10 g, the amount of carbohydrates that can produce glucose) was the same. Subsequent blood glucose tests were carried out 15, 30, 45, 60, 90, and 120 min after food consumption. The fasting glucose test and the subsequent blood glucose tests were all performed by Accu Chek performa (Roche Diagnostics). Participants consumed reference food (glucose solution) twice and each tested food (the three types of gummy candies) once.

Age, height, body weight, Body Mass Index (BMI), blood glucose, Glycemic Index (GI), and Glycemic Load (GL) were collected.

### 5.4. Texture Profile Analysis

The mechanical properties (hardness, springiness, cohesiveness, gumminess, chewiness, and resilience) of the gummy samples were analyzed at room temperature with a TA-plus Texture Analyzer (Stable Micro Systems Ltd., Surrey, UK). A cuboid shape of gummies (2 cm length × 2 cm width × 1.5 cm height) was applied for test. A circular probe (P/36R) with a diameter of 36 mm was used to perform compression. A constant crosshead speed of 1 mm/s was used for measurements, and the object was compressed twice to 80% of its initial height. Trigger force was set to 1 g, and the pre- and post-test speed were all set at 1 mm/s. The interval between the two compression cycles was 30 s.

### 5.5. Glucose Release in Gummies during In Vitro Digestion

Glucose release was assessed by the protocol of Khin et al. and Chen et al. using an in vitro digestion model [17,31]. SSF (simulated salivary fluid), SGF (simulated gastric fluid), and SIF (simulated intestinal fluid) were prepared according to the INFOGEST 2.0 method [17]. A swallowable bolus of 10 g of available carbohydrates was prepared from gummy samples. The swallowable gummies were mixed with 10 mL of SSF at a ratio of 1:1 (*w*/*w*) and incubated for 5 min at pH 7. The oral bolus was then diluted 1:1 (*v*/*v*) with SGF containing 1 mg/mL of gastric enzymes (pepsin and gastric lipase) and incubated for 1 h under agitation (200 rpm) at pH 3.0. The gastric chyme was then diluted 1:1 (*v*/*v*) with simulated intestinal fluid (SIF) containing 16 mg/mL of bile salts and 2 mg/mL of pancreatic enzymes, incubating at pH 7 with agitation of 200 rpm for another 2 h. In each phase, 100 µL of digestive juices was taken for glucose concentration analysis. Glucose concentration was measured using glucose oxidase method [18]. Glucose (5.57 mmol/L) was used as the standard solution. The released rate of glucose was calculated according to the following equation using UV-vis spectrophotometry at 520 nm:

$$\mathrm{Release}\;\mathrm{glucose}/\mathrm{available}\;\mathrm{CHO}\;(\%)\;=\frac{\mathrm{Amount}\;\mathrm{of}\;\mathrm{glucose}\;\mathrm{produced}\;\mathrm{during}\;\mathrm{digestion}}{\mathrm{Amount}\;\mathrm{of}\;\mathrm{available}\;\mathrm{carbohydrate}\;(g)}\;\times 100\%$$

### 5.6. Statistical Analysis

Statistical analysis was conducted using PASW^®^ Statistics Version 20.0 (SPSS, IBM^®^, Armonk, NY, USA). Descriptive statistics (mean ± standard deviation (SD)) were determined for all of the variables. All the subject acceptability scores were compared using a paired *t*-test (normally distributed) or Wilcoxon test (abnormally distributed). The area under the glucose response curve (AUC) above the fasting blood glucose levels was calculated using the Prism 8 software (GraphPad, San Diego, CA, USA). GI values of the sample food were calculated by averaging the tested food’s AUC over the AUC of glucose then multiplied by 100. The GL per gummy was calculated as the product of the GI and the weight in grams of the available carbohydrates in a gummy then divided by 100. The generalized estimation equation (GEE) was used to compare the effects of different fudge and time points on blood glucose concentration. Significant differences were determined at *p* < 0.05.

## Figures and Tables

**Table 1:** GEE analysis results of blood glucose concentration.

	B	SE	Wald Chi-Square	*p* Value
Glucose	reference			
CG	−0.238	0.105	5.169	0.023
P-SG	−0.401	0.106	14.263	<0.001
T-SG	−0.419	0.109	14.673	<0.001
0 min	reference			
15 min	0.948	0.093	102.358	<0.001
30 min	1.412	0.109	166.478	<0.001
45 min	0.842	0.064	168.192	<0.001
60 min	0.319	0.059	28.668	<0.001
90 min	−0.064	0.044	2.087	0.149
120 min	−0.153	0.041	13.658	<0.001

Sugar-free and 120 min were reference groups. GEE: Generalized estimation equation.

**Table 2:** Glycemic Index (GI) and Glycemic Load (GL) values and classifications of the three types of gummies.

	Glucose	CG	P-SG	T-SG
GI	100.00	81.9	54.1	49.9
GL	-	8.2	5.4	5.0

**Table 3:** Texture profile analysis parameters (TPA) of the three types of gummies (Mean ± SD).

	Hardness	Springiness	Cohesiveness	Gumminess	Chewiness	Resilience
CG	273.03 ± 14.97 ^a^	0.99 ± 0.01 ^a^	0.9 ± 0.01 ^a^	245.35 ± 13.32 ^a^	242.18 ± 13.78 ^a^	0.45 ± 0.02 ^a^
P-SG	431.2 ± 24.77 ^b^	0.93 ± 0.01 ^b^	0.79 ± 0.03 ^b^	341.26 ± 24.78 ^b^	317.13 ± 24.84 ^b^	0.36 ± 0.02 ^b^
T-SG	496.7 ± 28.2 ^c^	0.93 ±0.02 ^b^	0.82 ± 0.02 ^b^	405.01 ± 26.74 ^c^	374.87 ± 26.39 ^c^	0.36 ± 0.02 ^b^

^a, b, c^ Different letters mean significant differences (*p* < 0.05) of texture properties between gummy samples.

**Table 4:** The body index of subjects during experiment (Mean ± SD).

	Female (*n* = 12)	Male (*n* = 5)
Age (year)	28.6 ± 4.3	27.4 ± 3.1
Height (cm)	159.6 ± 5.8	172.0 ± 6.8
Body weight (kg)	54.5 ± 5.4	66.0 ± 8.5
BMI (kg/m2)	21.3 ± 1.3	22.2 ± 1.4
Blood glucose (mmol/L)	5.3 ± 0.3	5.2 ± 0.3

BMI: Body mass index.

**Table 5:** Macronutrient composition of each gummy sample.

	Glucose	CG	P-SG	T-SG
Carbohydrates * (%)	100	76.6	78	78.5
Protein (%)	0	4.7	3.9	3.8
Fat (%)	0	4	4	4
Water (%)	0	14.7	14.1	13.7

* Carbohydrates (%) consist of sugar and dietary fiber.

**Table 6:** Energy per gummy sample and reference glucose for each portion.

	Glucose	CG	P-SG	T-SG
Dosage (*n*)/test	-	1	2	2
Portion weight (g)/test	10	14.15	25.64	25.86
Calories (kcal)/test portion	40	47.1	65.9	59.36
Available carbohydrates (%) ^a^	100	71	39	39
Available carbohydrates (g)/test portion ^b^	10	10	10	10

^a^ Available carbohydrates (%) was acquired from WS/T 652-2019 Table A.1. ^b^ Available carbohydrates (g)/test portion was calculated by multiplying Portion weight (g)/test by Available carbohydrates (%).

## Data Availability

Data are contained within the article and supplementary materials.

## Supplementary Materials

The following supporting information can be downloaded at: https://www.mdpi.com/article/10.3390/gels8100642/s1, Table S1: Fabrication formulations of control and different test gummy samples; Table S2: Blood glucose levels (mmol/L) after consumption of a 10 g glucose bolus or three types of nutritional gummies providing equivalent amounts of available carbohydrates (Mean ± SD).
