# Supplementary material for: Intake of Sugar Substitute Gummy Candies Benefits the Glycemic Response in Healthy Adults: A Prospective Crossover Clinical Trial
Source: Gels. 2022 Oct 10;8(10):642. doi: 10.3390/gels8100642 (PMC9601933; doi:10.3390/gels8100642)
Supplement: Supplementary file 1 [file gels-08-00642-s001.zip › gels-1898219-supplementary.pdf]

Supplementary material

Intake of Sugar Substitute Gummy Candies Benefits the Glycemic Response in Healthy Adults: A Prospective Crossover Clinical Trial

Table S1. Fabrication formulations of control and different test gummy samples

	CG		P-SG		T-SG	
**Carbohydrates %**	Glucose syrup	41%	Maltitol	65 %	Maltitol	66%
	Sucrose	31%	Erythritol	8%	Erythritol	8%
	Concentrated apple juice	2%	Concentrated apple juice	2%	/	/
**Protein%**	Gelatin	6%	Gelatin	6%	Gelatin	6%
**Fat%**	DHA algal oil	4%	DHA algal oil	4%	DHA algal oil	4%

Note: Other ingredients include gum arabic, acid-treated starch, phospholipids, flavors, pigments, and acids, and the dosage is the same for each formula.

Table S2. Blood glucose levels (mmol/L) after consumption of a 10 g glucose bolus or three types of nutri-tional gummies providing equivalent amounts of available carbohydrates (Mean±SD)

**Time**	**Blood glucose (mean±SD）(mmol/L)**				**Significant difference**					
**(min)**	Glucose	CG	P-SG	T-SG	Glu vs CG	Glu vs P-SG	Glu vs T-SG	CG vs P-SG	CG vs T-SG	P-SG vs T-SG
**0**	5.29±0.32	5.24±0.03	5.28±0.33	5.24±0.33	ns	ns	ns	ns	ns	ns
**15**	7.02±0.57###	6.53±0.48###	5.62±0.36##	5.59±0.32##	*	***	***	***	***	ns
**30**	7.55±0.73###	6.98±0.75###	6.04±0.58###	5.90±0.35###	ns	***	***	***	***	ns
**45**	6.39±0.66###	5.96±0.58###	5.92±0.61##	5.93±0.51###	ns	ns	ns	ns	ns	ns
**60**	5.50±0.52	5.38±0.58	5.65±0.52#	5.63±0.57#	ns	ns	ns	ns	ns	ns
**90**	5.06±0.33#	5.09±0.38	5.28±0.35	5.28±0.41	ns	ns	ns	ns	ns	ns
**120**	4.99±0.33#	4.92±0.31##	5.15±0.32	5.33±0.40	ns	ns	*	ns	**	ns

Abbreviations: Glu, glucose，#, every times vs 0 min; #, $P < 0.05$; ##, $P < 0.01$; ###, $P < 0.001$; *, $P < 0.05$; **, $P < 0.01$; ***, $P < 0.001$
